# HEYL Regulates Neoangiogenesis Through Overexpression in Both Breast Tumor Epithelium and Endothelium

**DOI:** 10.3389/fonc.2020.581459

**Published:** 2021-01-15

**Authors:** Liangfeng Han, Preethi Korangath, Nguyen K. Nguyen, Adam Diehl, Soonweng Cho, Wei Wen Teo, Leslie Cope, Manfred Gessler, Lewis Romer, Saraswati Sukumar

**Affiliations:** ^1^ Department of Oncology, Johns Hopkins University School of Medicine, Baltimore, MD, United States; ^2^ Developmental Biochemistry, Comprehensive Cancer Center Mainfraken and Theodor-Boveri-Institute/Biocenter, University of Wurzburg, Wurzburg, Germany; ^3^ Department of Anesthesiology and Critical Care Medicine, Johns Hopkins University School of Medicine, Baltimore, MD, United States; ^4^ Department of Cell Biology, Johns Hopkins University School of Medicine, Baltimore, MD, United States; ^5^ Department of Biomedical Engineering, Johns Hopkins University School of Medicine, Baltimore, MD, United States; ^6^ Department of Pediatrics, Johns Hopkins University School of Medicine, Baltimore, MD, United States; ^7^ The Center for Cell Dynamics, Johns Hopkins University School of Medicine, Baltimore, MD, United States

**Keywords:** breast, cancer, endothelium, HEYL, notch, cytokines, epithelium

## Abstract

Blocking tumor angiogenesis is an appealing therapeutic strategy, but to date, success has been elusive. We previously identified HEYL, a downstream target of Notch signaling, as an overexpressed gene in both breast cancer cells and as a tumor endothelial marker, suggesting that HEYL overexpression in both compartments may contribute to neoangiogenesis. Carcinomas arising in double transgenic Her2-neu/HeyL mice showed higher tumor vessel density and significantly faster growth than tumors in parental Her2/neu mice. Providing mechanistic insight, microarray-based mRNA profiling of HS578T-tet-off-HEYL human breast cancer cells revealed upregulation of several angiogenic factors including CXCL1/2/3 upon HEYL expression, which was validated by RT-qPCR and protein array analysis. Upregulation of the cytokines CXCL1/2/3 occurred through direct binding of HEYL to their promoter sequences. We found that vessel growth and migration of human vascular endothelial cells (HUVECs) was promoted by conditioned medium from HS578T-tet-off-HEYL carcinoma cells, but was blocked by neutralizing antibodies against CXCL1/2/3. Supporting these findings, suppressing HEYL expression using shRNA in MDA-MB-231 cells significantly reduced tumor growth. In addition, suppressing the action of proangiogenic cytokines induced by HEYL using a small molecule inhibitor of the CXCl1/2/3 receptor, CXCR2, in combination with the anti-VEGF monoclonal antibody, bevacizumab, significantly reduced tumor growth of MDA-MB-231 xenografts. Thus, HEYL expression in tumor epithelium has a profound effect on the vascular microenvironment in promoting neoangiogenesis. Furthermore, we show that lack of HEYL expression in endothelial cells leads to defects in neoangiogenesis, both under normal physiological conditions and in cancer. Thus, HeyL-/- mice showed impaired vessel outgrowth in the neonatal retina, while the growth of mammary tumor cells E0771 was retarded in syngeneic HeyL-/- mice compared to wild type C57/Bl6 mice. Blocking HEYL’s angiogenesis-promoting function in both tumor cells and tumor-associated endothelium may enhance efficacy of therapy targeting the tumor vasculature in breast cancer.

## Introduction

Angiogenesis, a requisite for tumor growth, is the net result of a balance of angiogenic and antiangiogenic factors (1–3). Neoangiogenesis has remained a promising cancer therapeutic target for decades (4–6). However, the efficacy of anti-angiogenic therapies, mainly based on blocking VEGF function, has been limited in the clinical setting (7–12). This lack of therapeutic efficacy may be due to compensation by other angiogenic factors thereby resulting in resistance to anti-VEGF therapy (3, 13, 14), or simply due to a lack of sprouting angiogenesis in lymph nodes during early tumor dissemination (15, 16).

Devising effective anti-angiogenic therapy will depend on careful identification of novel factors on a genome-wide scale that promote angiogenesis in various cancer types. Using publicly available imaging and genomic data from the Cancer Genome Atlas GBM cohort, Rao et al. identified pathways associated with angiogenesis, tumor proliferation, and cerebral blood flow in glioblastoma (17). In another study, enhanced synthesis and secretion of members of the IL-8/GRO chemokine family was found to be associated with increased cell invasion and angiogenesis, and was implicated in metastatic progression and endocrine resistance of HER2-overexpressing breast carcinomas (18). In search of predictive biomarkers of improved survival in HER2-negative metastatic breast cancer in response to treatment with bevacizumab and paclitaxel, a predictive model of 13 genes and 5 clinical variates was deduced which identified patients with improved progression-free and overall survival (PFS and OS) (19). Elsewhere, expression of a 43-gene set showed the strongest correlation with the presence of endothelial “seed” genes in various cancers. It was proposed that this gene set could provide a metric for tumor angiogenesis and microvascular density (20).

Notch signaling activation has been reported in over 50% of breast cancers (21). Notch signaling in both epithelial and endothelial cells has also been shown to promote tumor angiogenesis (22–24). Notch pathway activation in cancer epithelial cells increased vascularization in the tumor microenvironment (25). In addition, Notch signaling in endothelial cells determines the fate of endothelial tip and stalk cells and maintains the functions and structures of the tumor vasculature (26). Given the significant impact of Notch signaling in tumor angiogenesis, numerous approaches aiming to target Notch signaling have been developed to inhibit tumorigenesis, and almost all the approaches have a dual effect on tumor epithelial and endothelial cells (27). Interestingly, it has been reported that Notch signaling contributes to resistance to bevacizumab, a humanized anti-human VEGF antibody, and that inhibiting Notch pathway enhances anti-VEGF efficacy (28). The known direct transcriptional targets of the Notch pathway are members of the HEY (hairy/enhancer-of-split related with YRPW motif) family, HEY1, HEY2, and HEYL (29, 30). However, HEYL is the only Notch downstream target gene that is associated with the expression of the Notch ligand, Jagged, in breast cancer tissues (31). We reported that HEYL is overexpressed in approximately 40% of breast cancer epithelial cells, and HEYL promoted breast cancer development by binding to TGFβ-activated Smads and inhibiting TGFβ activity (32). In 2004, we reported the discovery of HEYL as an overexpressed transcript through SAGE analysis of enriched populations of breast cancer endothelial cells from primary tumors (33). We also provided evidence that the expression of HEYL in tumor endothelial cells is potentially important for angiogenesis in breast cancer (33). Based on the literature and our own observation of HEYL expression in both the tumor endothelial and epithelial cells, we proposed that of the downstream effectors of Notch, among the three members of the HEY family, HEYL may be the primary mediator of Notch pathway action to induce tumor angiogenesis in breast cancer.

In this paper, using engineered mouse and human model systems, we investigated whether HEYL is a key regulator of Notch-mediated angiogenesis in both epithelial and endothelial compartments in breast cancer. Here, we show that HEYL expressed in tumor epithelial cells increased expression of multiple angiogenic factors and promoted neoangiogenesis, while HEYL expression in endothelial cells appears to promote invasive growth behavior of endothelial cells. Moreover, targeting two key molecules important for angiogenesis, CXCR2 along with VEGF achieved improved therapeutic endpoints. Thus, inhibition of angiogenic factors induced by HEYL and VEGF using combinatorial regimens may result in more clinically effective anti-angiogenic therapy.

## Methods

### Establishment of HS578T-tet-off HEYL Inducible Cell Lines

All the cell lines used in this study were authenticated within the last year or were used in their early passages after receipt from ATCC. Myc-tagged HEYL was cloned into a promoterless pcDNA3-hygro vector fragment. The final vector, pcDNA3-hygro-HEYL, was transfected into breast cancer cell line HS578T with tTA-IRES-Neo vector. After selection, individual clones were picked and cultured in 125 ug/ml hygromycin B, 100 ug/ml G418 and 10 ng/ml doxycycline for expansion.

### Detecting Cytokines in the Supernatant of HEYL-Inducible Cells

Antibody arrays (RayBiotech, Human Cytokine Array AAH-CYT-1-2) were used to detect cytokines in the supernatant of HS578T-tet-off-HEYL cells, under induced- (no doxycycline) and uninduced- (with doxycycline) conditions. The arrays were blocked for 30 min and incubated at 4°C overnight with 1ml supernatant of HEYL-inducible cells under uninduced and induced condition for 6 h. After washing, a biotinylated antibody cocktail provided by the manufacturer was added to the array and incubated at room temperature for 2 h. After washing, the arrays were incubated with HRP-Streptavidin at room temperature for 2 h. The arrays were washed again and incubated with detection buffer mixture at room temperature for 2 min. Finally, the chemiluminescent signals were detected.

### Establishment of MDA-MB-231 Cells With shRNA Mediated Knockdown of HEYL

Retroviral vectors expressing scramble shRNA or 2 shRNAs targeting HEYL (shRNA1: ATGGGTCTCTGAAATCACTGAA, shRNA2: AGACTTGCATCTTGTGTTTCTA) were purchased from Openbiosystems. Retroviral packaging was performed in 293T cells. The viral supernatant was used to infect MDA-MB-231 cells which were selected with 2 ug/ml puromycin. shRNA1 targets the very C-terminal end of HEYL coding region, which does not disrupt HEYL function. A myc-tagged HEYL lacking the HEYL target region of shRNA1 (hence shRNA1-resistant) was cloned into PLHCX retroviral vector. The retroviral supernatant was collected as above and used to infect MDA-MB-231 cells. Infected cells were selected in medium containing 250 ug/ml hygromycin B.

### Generation and Characterization of MMTV-HeyL/Her2-neu Double Transgenic Mice

All animal experiments were conducted with approval from the Johns Hopkins University Animal Care and Use Committee, and performed according to their guidelines. Transgenic FVB/N, MMTV-HeyL (32) were crossed with FVB/N, MMTV-Her2-neu mice that overexpress wild type rat HER2/neu (34, 35) in the mammary gland to generate MMTV-HeyL/Her2-neu double transgenic mice. Virgin Her2-neu or HeyL/Her2-neu double transgenic mice (n=22 in each group) were examined for mammary tumor development twice a week. Tumor size was measured using electronic calipers and calculated as following: volume=0.5236*L*W*W.

### Microarray Analysis of Gene Expression Using tet-off HEYL-Inducible Cells

RNeasy mini kit (Qiagen) was used to extract RNA at 6 and 24 h after doxycyline withdrawal from HS578Ttet-off-HEYL cells. Following quality control, the RNA was hybridized to Affymetrix human U133 2.0 arrays. The data was analyzed by the Johns Hopkins Microarray Core.

### Real-Time RT-qPCR

RNA was extracted using RNeasy mini kit and treated with DNAse I to remove genomic DNA contamination (32). One microgram total RNA was used for reverse transcription with MuMLV-reverse transcriptase (Promega). 0.25 ug cDNA was used as template and quantitative PCR was performed with Quantitect SYBR Green PCR kit (Qiagen). The primer sequences are available on request. mRNA levels were expressed as delta Ct values relative to 36B4, a ribosomal gene.

### Vascular Characterization–Quantitation of CD31-Stained Microvessels

Methods were followed as described (36). Formalin fixed paraffin-embedded sections of 2 mammary glands each from 5 mice were stained using the anti-CD31 antibody (Dianova). A 1:40 dilution of the antibody was used to stain the sections overnight. Diluted biotinylated anti-rat IgG (Vectastain kit) was added to the sections and incubated for 30 min. Vectastain ABC reagent (Vector) and 3, 30-diaminobenzamidine (DAB) was then used for color development. The vessel number, cumulative circumferential vessel length and cumulative vessel area were quantified using MetaVue software. For more details, refer to **Supplementary Methods**.

### HUVEC Branching Network Formation, Migration, and Invasion Assay

Methods described in (37) were followed with modifications. For the migration assay, 2x10^4^ HUVEC cells were added in the upper chamber, and 750 ul complete, uninduced or induced HS578T-tet-off-HEYL conditioned medium (CM) was added to bottom chamber. For the invasion assay, 1.5x10^4^ HUVEC cells were added in the upper chamber, and 750 ul complete EGM2 medium (Lonza) was added to bottom chamber. For both migration and invasion assays, the cells in the upper chamber were removed 16 h later with a cotton tip, and the migrated cells at the bottom of the membrane were fixed and stained by crystal violet. The number of cells on the membrane was manually counted for each well and averaged across the three wells for each treatment condition. For the branched network formation assay, 30 ul Matrigel was added in the wells in 96-well plates. After the gel was solidified, 2x10^4^ HUVEC cells in 100 ul complete EGM2 media, uninduced or induced CM were added onto the gel. Sixteen hours later, the cells were fixed. For each well, five random 10x fields were chosen for assessment of end points. A branch point cell was defined as any cell with 3 or more connections to neighboring cells. The number of branch point cells was calculated for each field, and the average number of branch point cells per field was calculated for each condition. This average number was compared across conditions. To test the effects of antiangiogenic antibodies or drugs, branched network formation assays were performed. Four micrograms per milliliter anti-CXCL1/2/3, and 5 nM SB265610 was added to the CM from induced cells. Each experiment was repeated at least 3 times.

### Tumor Xenografts and Treatment in Mice

To study the effects of HEYL depletion or overexpression, 1x10^6^ MDA-MB-231-scr or MDA-MB-231-HEYL–shRNA expressing cells were injected into the mammary fat pad (mfp) of Balb/c nu/nu mice. Syngeneic C57/Bl6 HeyL+/+ and HeyL-/- mice received mfp injections of E0771 mouse mammary tumor cells (5x10^5^). For testing therapies, MDA-MB-231 (5x10^5^) cells were injected subcutaneous (sc) into flanks of Balb/c nu/nu mice. Mice were treated intraperitoneally (ip) with vehicle, the CXCR2 inhibitor SB265610 (2 mg/kg/daily), the VEGF-inhibitor bevacizumab (10 mg/kg/biweekly) or a combination of SB265610 plus bevacizumab for 3 weeks. Six to nine mice/group were used in each experiment.

### Retinal Vessel Staining

The techniques were essentially as described in (38). Six-day old Heyl +/+ and HeyL -/- mouse pups (littermates from the same cage) were used for comparison. A freshly prepared stock (3 mg/ml) of 5′-bromo-2′ deoxyuridine (BrdU, Invitrogen, cat. no. B23151) was used each time; 300 μg of BrdU was injected intraperitoneally (i.p.) into the 6-day-old pups. 2.5 h later the mice were euthanized and the eyes fixed in 4% paraformaldehyde (PFA) at 4°C overnight, washed in PBS and retina was dissected out.

The retina was flattened with either four or five incisions radially around the optic nerve. Therefore, each retina had four or five wedges extending from the optic nerve along which measurements of retinal vessel migration distance could be made. After blocking/permeabilization, the retina was stained for 2 h in PBS containing biotinylated isolectin B4 (Vector Labs 1:50) (39, 40). Eleven retinas from 7 HeyL-/- mice and 8 retinas from 5 HeyL+/+ mice were examined. 4x images (2738 um x 2086 um) were taken of each of the 4 or 5 retinal wedges and Image J software was used to quantify the length of a line segment bisecting the wedge and extending from the outer margin of the optic nerve to the border of the vascular network. The average length from the optic nerve to the vascular front was calculated for the HeyL-/- mice and the HeyL+/+ mice and the two were compared using 2-tailed Student’s T test. Detailed methods for BrdU quantification and processing and staining of retina are provided in **Supplementary Methods**.

### *In Vitro* 3-D Fibrin Gel Culture

This assay was performed as reported (41). One million HUVEC cells were incubated with Cytodex 3 beads in EGM2 media overnight. 25 beads plus 200 ul of 2.5 mg/ml fibrinogen were added to each well of 48-well plate that contained 50U/ml thrombin. After the gels were solidified, 600 ul EGM2 media plus 60 ul conditioned media (CM) from primary human lung fibroblasts was added. Sprout length was measured using Image J imaging software.

### *In Vivo* Matrigel Plug Assay

Methods described in (42) was followed. Five hundred microliter growth factor-reduced Matrigel (#354234, Corning) plugs containing 150 ng/ml bFGF were injected sc into the ventral flank of wild type HeyL +/+ and HeyL -/- mice (n=5). On day 10 the plugs were removed, fixed in 10% formalin, sectioned and stained with Masson’s Trichrome to detect ECM of blood vessels.

### Statistical Tests

Each experiment was performed at least three times. Significance between different groups studied for angiogenesis was calculated using Welch’s corrected T-test, and Bonferroni correction was applied as multiple test correction where appropriate. Significance of differences in xenograft growth in mice was calculated by ANOVA 2-way comparisons with Bonferroni correction for comparison of effects between treatment groups with time. GraphPad Prizm software was used. *P<0.05, **P<0.01, ***P<0.001.

## Results

### HeyL Transgenic Mice Show Characteristics of Increased Angiogenesis in the Mammary Gland and Tumors

**Figure 1:**
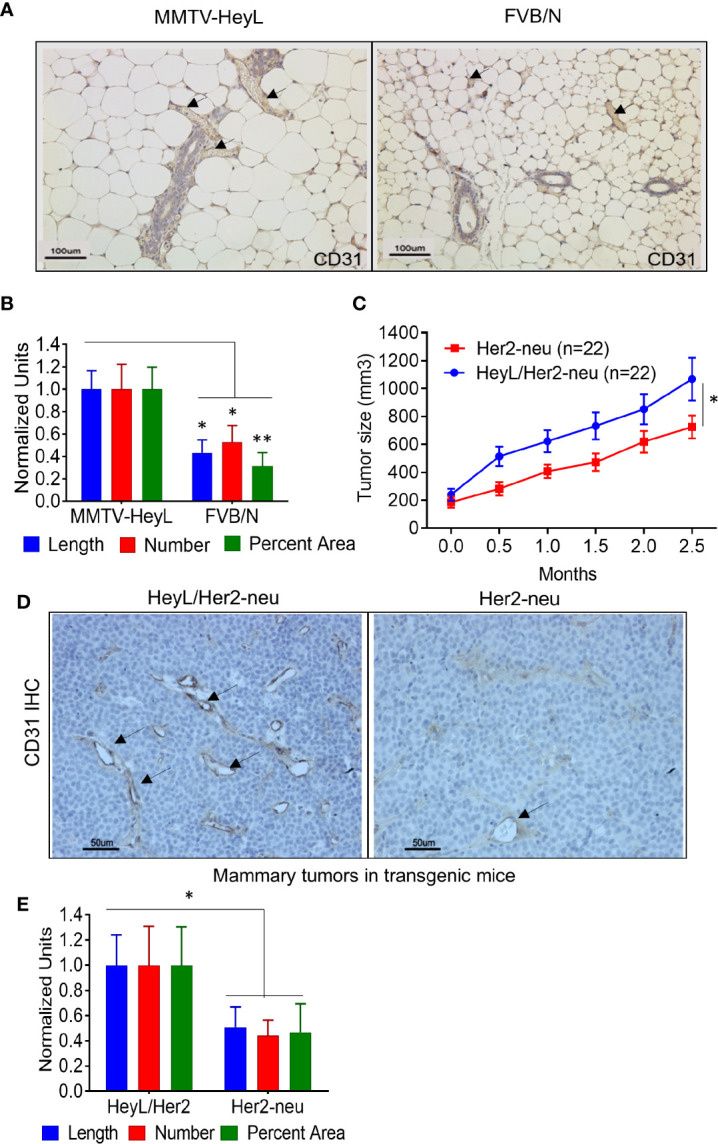
HeyL promotes angiogenesis in transgenic mice. **(A)** Anti-CD31 IHC analysis of blood vessels in mammary glands of 13 week-old virgin, MMTV-HeyL transgenic mice compared to parental FVB/N mice (n=4 mice/group). **(B)** Number of blood vessels, vessel length and average area occupied by blood vessels in mammary glands from MMTV-HeyL and parental FVB/N mice, analyzed in (A). **(C)** Mammary tumors arising spontaneously in MMTV-HeyL/Her2-neu are larger compared to those in MMTV-Her2-neu mice (n=22/group). Measurement was initiated after detection of palpable tumors. **(D)** Anti-CD31 IHC analysis of blood vessels in mammary tumors in MMTV-HeyL/Her2-neu compared to MMTV-Her2-neu mice from (C), (n=6 mice each group) **(E)**. Number of blood vessels, vessel length and average area occupied by blood vessels in mammary tumors in MMTV-HeyL/Her2-neu mice compared to MMTV-Her2-neu mice from (D) *P < 0.05; **< 0.05.

We have previously reported the finding that 24% of the multiparous MMTV-HeyL transgenic mice developed mammary tumors at 13–20 months of age (32). To examine if HeyL expression in epithelial cells contributes to neoangiogenesis, we compared characteristics of blood vessel growth in the mammary gland of 13-week old virgin MMTV-Heyl transgenic mice to the mammary gland of parental FVB/N mice. Using an anti-CD31 antibody, immunohistochemical (IHC) analysis of mammary tissue from MMTV-HeyL transgenic mice showed significantly higher vessel density in the mammary gland (**Figure 1A**), vessel length, number and percent area (**Figure 1B**) compared to wild-type FVB/N mice. To test if this enhanced blood vessel formation caused by the HeyL transgene affects tumor growth, MMTV-HeyL mice were crossed with MMTV-Her2-neu mice that overexpress wild type HER2/neu proto-oncogenes. The HER2 proto-oncogene has been reported to be overexpressed and amplified in about 15 to 20% of breast cancers (43). For the subtype of HER2-positive breast cancers, a variety of anti-HER2 antibody-based therapies and novel tyrosine kinase inhibitors have been developed and have achieved significant anti-tumor effects (44). It has also been reported that the crosstalk between HER2 activity and Notch signaling contributes to tumor resistance to anti-HER2 monoclonal antibody, trastuzumab (45). Mammary tumors arising in the MMTV-Heyl/Her2-neu mice grew significantly faster compared to those in the MMTV-Her2-neu transgenic mice (**Figure 1C**). By IHC analysis, MMTV-HeyL/Her2-neu transgenic mouse tumors had a higher density of blood vessels (**Figure 1D**), vessel length, number and percent area (**Figure 1E**) compared to mammary tumors arising in MMTV-Her2-neu mice.

### HEYL Induces the Expression of Multiple Angiogenic Factors

**Figure 2:**
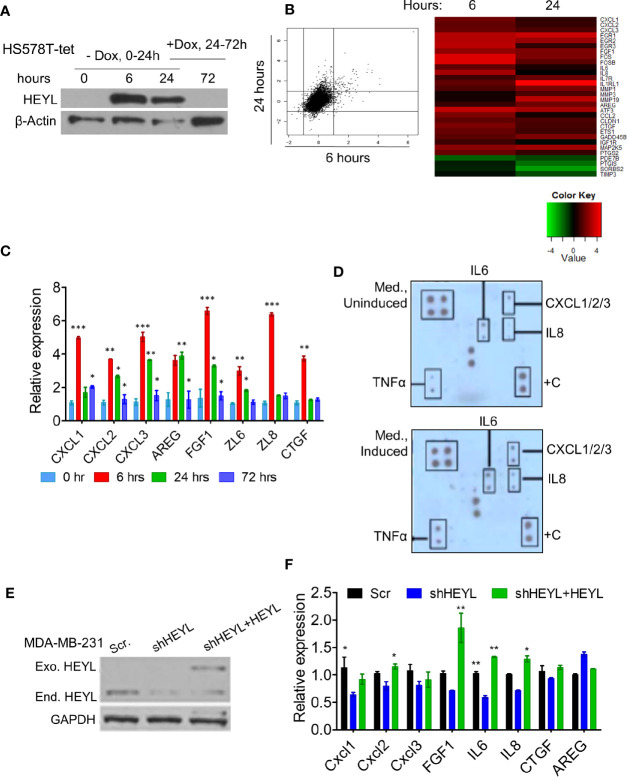
HEYL controls the expression of multiple angiogenic factors. **(A)** Western blot analysis of Myc-tagged HEYL expression in HS578T-tet-off-HEYL at 0, 6, and 24 h after doxycyline (dox) withdrawal. At the 24 h time point, Dox was re-added, and Myc-tagged HEYL expression was examined 48 h later (72 h after initiation of the experiment). **(B)** A scatter plot (showing log2-fold change) and heatmap show genes with expression changes at 6 and 24 h after HEYL induction relative to 0 h cells (uninduced cells) (also listed in **Supplementary Table 1**). **(C)** RT-qPCR validation of expression of major cytokines in the HS578T-tet-off-HEYL-inducible cells in (B) at 0, 6, 24, and at the 72 h time point (48h after re-induction of HEYL expression). mRNA expression levels relative to 36B4, a ribosomal gene, are shown. **(D)** Antibody array-based detection of cytokines in conditioned medium from HS578T-tet-off-HEYL-induced and –uninduced cells (6h); +C are quality controls **(E)** Western blot analysis of HEYL in MDA-MB-231-shRNA cells and re-expression after introduction of shRNA-resistant, Myc-tagged HEYL. **(F)** qPCR of cytokine expression in the MDA-MB-231 cell panel in (E). mRNA expression levels relative to 36B4, a ribosomal gene, are shown. *P < 0.05; **< 0.05; ***< 0.001.

HEYL is a basic Helix-Loop-Helix transcriptional factor, but the genes that it regulates are not known. To investigate the gene expression profile induced by HEYL, we generated a HS578T-tet-off-HEYL cell line that expresses myc-tagged HEYL after doxycycline withdrawal. After 24 h of gene induction/dox withdrawal, reintroduction of doxycycline to the medium for an additional 48 h (72 h from 0 time point) resulted in decreased HEYL expression (**Figure 2A**). Microarray analysis was performed using RNA extracted from these cells at 0, 6, and 24 h after induction. The data showed that upon HEYL-induction only approximately 100 genes underwent changes in expression that were higher than 2-fold (**Figure 2B**, **Supplementary Table 1**). Interestingly, many of these genes were secreted proteins, and included well-known angiogenic cytokines such as CXCL1, 2, and 3, IL6, IL8, and FGF1 (**Figure 2B**). Consistent with the microarray data, by RT-qPCR, expression of these genes was higher at 6 and 24 h following HEYL induction compared to baseline (**Figure 2C**). When doxycycline was added back to the medium 24 h post-HEYL induction, expression of these genes was reduced (**Figure 2C**). To determine changes in protein expression, conditioned medium (CM) from HS578T-tet-off-HEYL cells with, or without HEYL induction for 6 h was analyzed by protein array. In a pattern consistent with the gene expression data, increased levels of angiogenic cytokines were found in the media from cells with induced HEYL expression (**Figure 2D**). We tested the effect of HEYL expression on cytokine gene expression in a second breast cancer cell line, MDA-MB-231, with shRNAs mediating downregulation of endogenous HEYL (**Figure 2E**). Compared to the MDA-MB-231 cells expressing scramble-shRNA, MDA-MB-231 cells with HEYL-shRNA expression showed reduced mRNA expression of these cytokines (**Figure 2F**). Conversely, also shown in the western blot (**Figure 2E**), when an exogenous HEYL (that was not targeted by the shRNA) was expressed in MD-MB-231 cells, cytokine expression increased significantly or showed the same trend (**Figure 2F**). The results in both model systems, using a tet-inducible HS578T system and using downregulation of HEYL with shRNA, followed by re-expression of HEYL in MDA-MB-231 cells strongly suggested that HEYL may, directly or indirectly, regulate the expression of a number of cytokines that have been previously implicated in neoangiogenesis.

To determine if HEYL exerts direct transcriptional control on the expression of the cytokine genes, we searched for HEYL consensus DNA-binding sites from previously published HEYL ChIP-seq data on HEYL-expressing HEK293 cells (46). Heisig et al. reported that HEYL bound strongly to the promoter regions of CXCL1, 2, and 3, while HEY1 and HEY2 displayed much lower binding affinity to these sites (46). We performed ChIP assays to test whether HEYL bound to the promoter regions of CXCL1, 2, or 3 in HS578T-tet-off-HEYL cells. Gene loci for these three chemokines are located on the same chromosome, separated from one another by 60–170 kb. HEYL did not bind to all of the sites predicted by the ChIP-seq data (46). As shown by PCR analysis of the immunoprecipitated protein-DNA complex in HS578T cells, HEYL bound to the following regions: 2 kb upstream of the CXCL1 transcription start site (TSS), 2 kb upstream of the CXCL2 TSS, close to both the TSS for CXCL2 and for CXCL3, and 1.4 kb upstream of the CXCL3 TSS (**Supplementary Figure 1**). These data supported the notion that HEYL is a direct regulator of CXCL1, -2 and -3. We hypothesized that HEYL expression in the tumor cells, in all likelihood, promotes angiogenesis through upregulation and expression of these and other chemokines.

**Figure 3:**
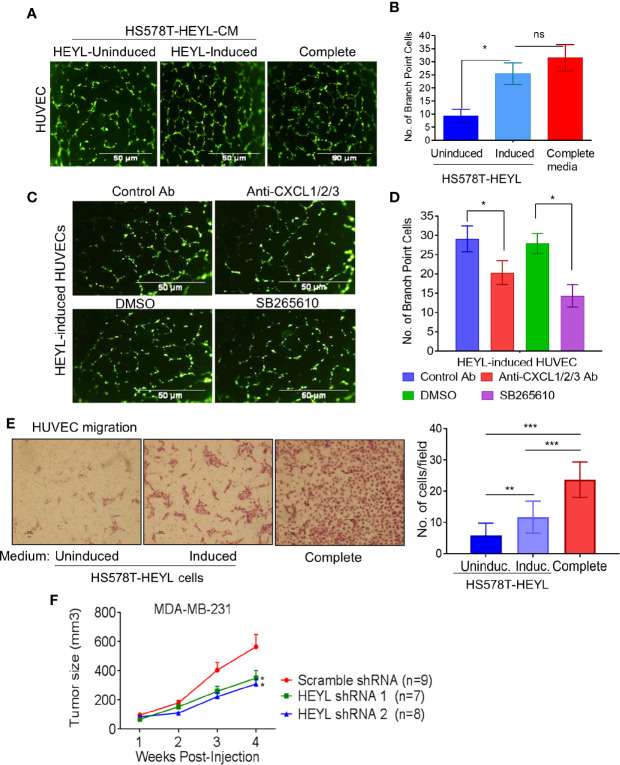
HEYL functionally regulates angiogenesis *in vitro* and supports tumor growth *in vivo*. **(A)** Branched network formation on Matrigel of HUVEC cells incubated with complete EGM2 medium, or conditioned medium from HS578T-tet-off-HEYL-induced, or –uninduced cells. **(B)** The quantification of cells with more than 3 branching sites in (A). **(C)** Antibodies that neutralize CXCX1/2/3, or a CXCR2 inhibitor, SB265610 (SB) reduced HUVEC branched network formation. **(D)** The quantification of cells with more than 3 branching sites in (C). **(E)** Cell migration assay with HS578T-tet-off-HEYL cell- induced or uninduced conditioned media. Panels show stained HUVECs that have migrated through a membrane in a Boyden chamber assay. **(F)** Growth rate of xenografts in nu/nu mice of MDA-MB-231 with scr-shRNA (n=9) or shRNA1 (n=7) and shRNA2 (n=8) targeting HEYL. *P < 0.05; **< 0.05; ***< 0.001.

We tested this concept in cultured human vascular endothelial cells (HUVEC). HUVEC cultured on Matrigel using CM from induced HS578T-tet-off-HEYL cells formed more branched network like structures than HUVEC exposed to CM from uninduced cells (**Figures 3A, B**). Neutralizing antibodies against CXCL1, -2, and -3 inhibited HUVEC branched network formation [**Figures 3C** (upper panels), **D**]. Since CXCL1, -2, -3 and IL8 all bind to the CXCR2 receptor (47), a potent CXCR2 receptor blocker, SB265610, was also tested. Branched network formation in HUVEC cells was inhibited when SB265610 was added to CM from induced-HS578T-tet-off-HEYL cells [**Figures 3C** (lower panels), **D**]. Additionally, HUVECs migrated more rapidly toward CM from induced cells than uninduced cells (**Figure 3E**). These data indicate that angiogenic chemokines and/or cytokines present in HEYL-induced CM promoted angiogenesis *in vitro*. Next, we examined HEYL’s ability to promote growth in xenografts of human breast cancer cells. Immunodeficient mice received injections (mfp) of MDA-MB-231 cells expressing scr-shRNA, or shRNA1 or shRNA 2 that target HEYL. MDA-MB-231-scr cells grew significantly faster (*p<0.05) than MDA-MB-231-HEYLshRNA cells (**Figure 3F**).

### HEYL Expression in Tumor Endothelial Cells Promotes Cell Invasion

**Figure 4:**
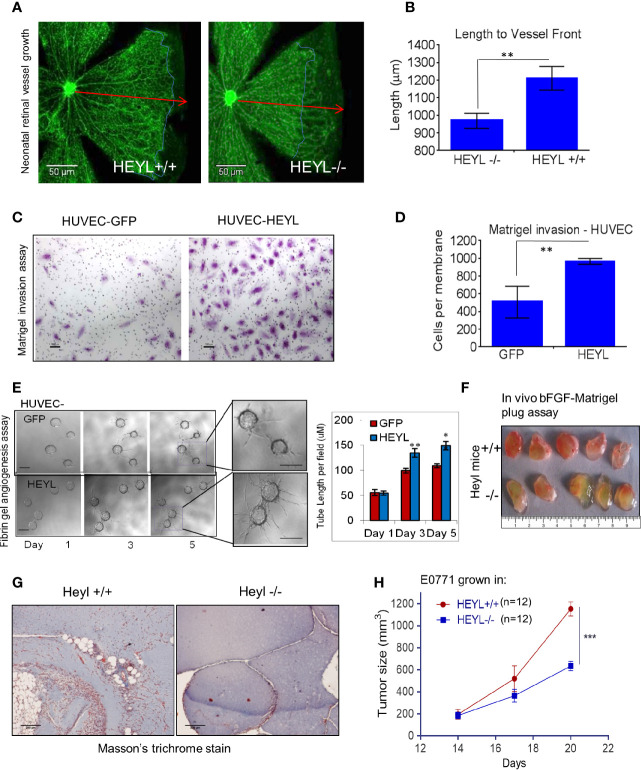
HEYL expression in endothelial cells promotes endothelial cell invasion. **(A)** Retinal blood vessel growth in neonatal (day 6) retina in HeyL+/+ mice extends farther than in HeyL-/- mice. The blue lines in the figure indicate the extent of outgrowth of the blood vessels while the red arrows define the length achieved in retina of wild type mice. **(B)** A significant difference is observed in the length of the blood vessels from center to periphery of the retina of 6-day old HeyL+/+ and Heyl-/- mouse pups. **(C)** Matrigel invasion assay of HUVEC-HEYL or HUVEC-GFP cells. **(D)** Quantification of the number of the invaded cells in a Matrigel invasion assay shows HUVEC-HEYL cells migrate 2X faster than control HUVEC–GFP cells. **(E)** (Left panel) 3-D fibrin gel invasion assay was used to measure growth of sprouts of HUVEC-GFP or HUVEC-HEYL cells at day 1, 3 and 5. Right panel: The average sprout length was measured in the two groups. Quantification of three determinations is shown. **(F)** Matrigel plugs containing bFGF examined 10 days after sc implantation in wild type HeyL+/+ and HeyL -/- mice show different extent of mouse blood vessel infiltration (n=5 mice per group). **(G)** Sections of matrigel from (F) were stained with Masson’s Trichrome to visualize ECM surrounding blood vessels show greater infiltration in HeyL-/- mice compared to HeyL+/+ mice (n=5 per group). **(H)** Impaired growth of mouse mammary tumor, E0771, in the mammary fat pad of syngeneic HeyL-/- mice compared to HeyL+/+ mice (n =12 per group). *P < 0.05; **< 0.05; ***< 0.001.

We had previously reported that serial analysis of gene expression (SAGE) profiles showed that HEYL is upregulated 20-fold in tumor endothelial cells of breast cancer compared to EC from normal breast (33, 48). We also showed that HEYL promoted HUVEC proliferation and survival (33). These data strongly suggested that the differential expression of HEYL in another tumor compartment, i.e., endothelial cells, may support tumor cell growth. Therefore, to examine the effect of the presence or absence of HEYL in endothelial cells on angiogenesis *in vivo*, we studied neonatal mouse retinal blood vessel growth in wild type (WT) and HeyL-/- mice (49, 50). At postnatal day 6, retinal vessels in WT mice reached the periphery (red arrows, **Figures 4A, B**). However, in HeyL-/- mice of the same age, incomplete vessel growth and invasion was observed (blue outline of margins, **Figures 4A, B**). This finding could be attributed to a slow rate of growth of the retinal vessels. No differences were observed in BrdU incorporation in the endothelial cells of WT compared to HeyL-/- mice (**Supplementary Figure 2**), suggesting that cell invasion, not proliferation, accounted for differences in retinal angiogenesis. Therefore, the expression of HEYL in endothelial or the cells in their microenvironment may assist vessel invasion into normal anatomical areas. Similarly, invasion through Matrigel in Boyden chambers of HUVEC cells expressing GFP was less frequent than HUVEC cells expressing HEYL (**Figures 4C, D**). Also, in 3-D fibrin gel cultures, HUVEC-HEYL-cells showed faster invasion with longer sprout lengths (**Figure 4E**). These findings suggested that loss of HEYL might lead to defects in neoangiogenesis. Direct evidence was sought for HeyL’s involvement in mouse tumor angiogenesis and growth *in vivo*. Matrigel plugs containing bFGF were injected subcutaneously (sc) into HeyL+/+ and HeyL-/- mice. Gel plugs retrieved from HeyL knockout mice had fewer blood vessels and a paler appearance compared to plugs from HeyL+/+ mice (**Figure 4F**). Sections of plugs stained with Masson’s trichrome to visualize vessel ECM supported these observations (**Figure 4G**). Furthermore, HeyL+/+ mice supported the growth of syngeneic E0771 mouse mammary tumors better than HeyL-/- mice (**Figure 4H**). Tumors grew significantly larger in HeyL+/+ mice than in HeyL-/- mice. Since these mice lack HEYL expression in all cell types, we do not know whether the impeded tumor growth is due of HeyL loss in endothelial cells or in any other cell population.

### Blocking VEGF and CXCR2 Signaling Enhanced Antiangiogenic Effects of Bevacizumab

**Figure 5:**
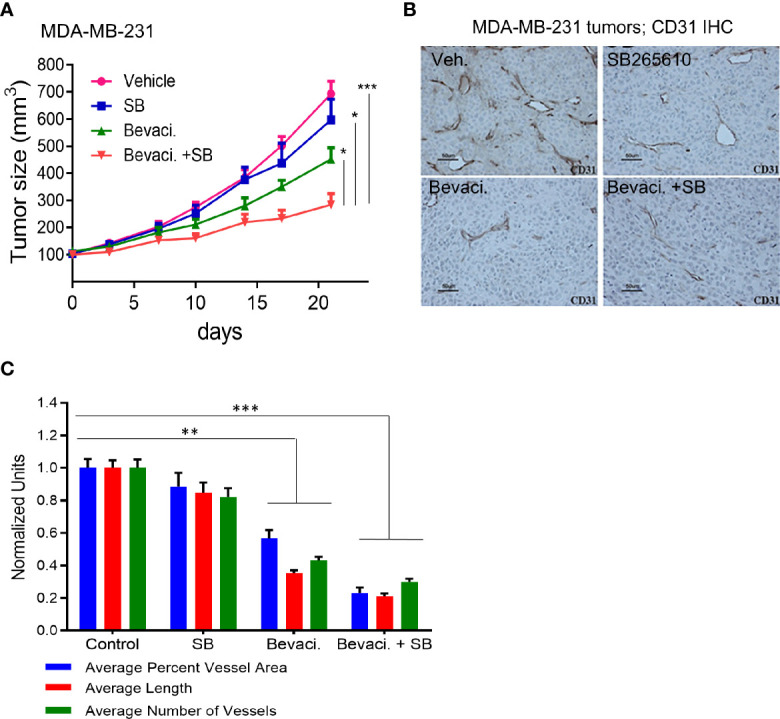
Targeting Notch pathway and VEGFR2 inhibits tumor growth. **(A)** Growth of sc xenografts of MDA-MB-231 cells in immunodeficient mice treated with vehicle (n=8), SB265610 (n=7), bevacizumab (n=9) and SB265610 plus bevacizumab (n=9). **(B)** Anti-CD31 IHC analysis of blood vessels in MDA-MB-231 xenografts shows significant reduction of tumor size in mice treated with bevacizumab and a combination of bevacizumab plus SB265610 compared to other groups. **(C)** Quantification of number of blood vessels, vessel length and average area occupied by blood vessels in mice (B) treated with single agents or combination of bevacizumab plus SB265610 compared to vehicle control mice. *P < 0.05; **< 0.05; ***< 0.001.

The evidence presented thus far suggested that an epithelial–endothelial crosstalk elicits tumor-specific angiogenesis. This provides a therapeutic opportunity for combined targeting of both compartments to interrupt signaling leading to neo-angiogenesis. The efficacy of bevacizumab, a humanized anti-human VEGF antibody in breast cancer was not high enough to warrant its use as a single agent (7, 8). This lack of efficacy could be attributed to the fact that many other angiogenic factors are expressed in breast cancer. For example, CXCR2 activation upon cytokine binding leads to VEGFR2 transactivation either directly through CXCR2 interaction with VEGFR2, or indirectly through NFĸB-dependent autocrine activation of VEGFR2 receptors (51). Since HEYL increased expression of CXCR2 receptor ligands (CXCL1, 2, and 3, and IL8) (**Figure 2**), we tested the combined antitumor effects of known anti-CXCR2 and anti-VEGF agents using MDA-MB-231 cells with high endogenous expression of HEYL. Treatment with the CXCR2 inhibitor, SB265610, plus the anti-human VEGF antibody, bevacizumab, significantly reduced tumor growth of MDA-MB-231 xenografts compared to bevacizumab or SB alone (**Figure 5A**). Tumor vessel density was decreased by treatment with either SB265610 or bevacizumab alone as compared with control, while the combined treatment (bevacizumab +SB) was even more effective (**Figures 5B, C**). These results suggested that HEYL, through its action on CXCL1, 2, 3 and IL8, contributed substantially to promoting tumor growth, likely through its effects on the tumor endothelial cells. Thus, reduction of tumor size was achieved by using a combination of specific inhibitors of the cytokine receptor and VEGF.

## Discussion

Tumor angiogenesis is a complex process involving cooperative interactions between carcinoma cells and their microenvironment. In this paper, we present evidence that overexpression of HEYL in breast carcinoma cells elicits a response in both tumor epithelial and endothelial cells that promotes angiogenesis.

Identification of angiogenic factors, other than VEGF, has remained a critical quest in cancer therapy. The Notch pathway has been reported to function in both tumor epithelial and endothelial cells to support tumor growth and angiogenesis. Various therapies targeting the Notch pathway including Notch receptor/ligand monoclonal antibody and inhibitors of γ-secretase, that is an enzyme necessary for Notch receptor cleavage and signal transduction, have been developed to inhibit tumor growth and showed promising anti-angiogenesis effects (27). HEYL is the only Notch downstream target gene that correlated to the expression of the Notch ligand, Jagged, in breast cancer tissues (31). High HEYL expression was found to be present in about 40% of breast cancers—a pattern consistent with enhanced Notch signaling in breast cancer (32, 33). We have also shown previously that HEYL is a direct transcriptional target of Notch in breast cancer cells (32).

Our functional assays indicate an important role for epithelial HEYL expression in the promotion of tumor angiogenesis. We have shown that HEYL increased the expression of several angiogenic cytokines (**Figure 2**) and enhanced angiogenesis and tumor growth by its action in both epithelial (**Figure 3**) and endothelial cells (**Figure 4**). CXCR2 is a receptor for CXCL1, 2, and 3, and IL8 that has been shown to have a profound effect on tumor epithelial interactions with stromal cells (52). Combined treatment of MDA-MB-231 xenografts using a CXCR2 inhibitor, SB265610, and an anti-VEGF antibody, bevacizumab, showed significant anti-tumor effects (**Figure 5**) both on size of the tumor and reduction in neoangiogenesis. Thus, our work revealed novel anti-angiogenesis strategies that might target tumors with an active Notch-HEYL-angiogenic factor axis using a CXCR2-inhibitor combined with bevacizumab. Our results are consistent with previous reports in which an enhanced anti-angiogenesis effect was achieved by using bevacizumab in combination with DBZ, a small molecule inhibitor of γ-secretase to inhibit Notch signaling (28). Collectively, these data also suggest that high HEYL expression in tumors may help identify patients who may be amenable to anti-Notch or anti-CXCR2-targeted therapies. In addition, we found that HEYL expression in both tumor epithelial (**Figure 3**) and endothelial cells (**Figure 4**) has functional consequences. Overexpression of HEYL in HUVEC promoted cell invasion. The loss of HEYL expression in endothelial cells reduced endothelial cell invasion *in vitro*. Absence of HEYL in the HEYL-/- mice impaired physiological retina blood vessel development and tumor growth of syngeneic cancer cells (**Figure 4**). These findings are in concordance with our previous observations that HEYL overexpression augmented proliferation in human mammary microvascular endothelial cells, and also was protective against apoptosis in HUVEC (33). However, a direct function for HEYL in endothelial cells during neoangiogenesis still remains to be substantiated. In HEYL-/- mouse retina, the observed phenotype of retarded retinal vessel outgrowth could be attributed to non-endothelial cells in the retina such as macrophages and pericytes, as well as to endothelial cells. Similarly, slower growth of syngeneic E0771 tumor cells in HEYL-/- mice may occur due to lack of HEYL in other cell types in the tumor microenvironment or due to distant effects.

**Figure 6:**
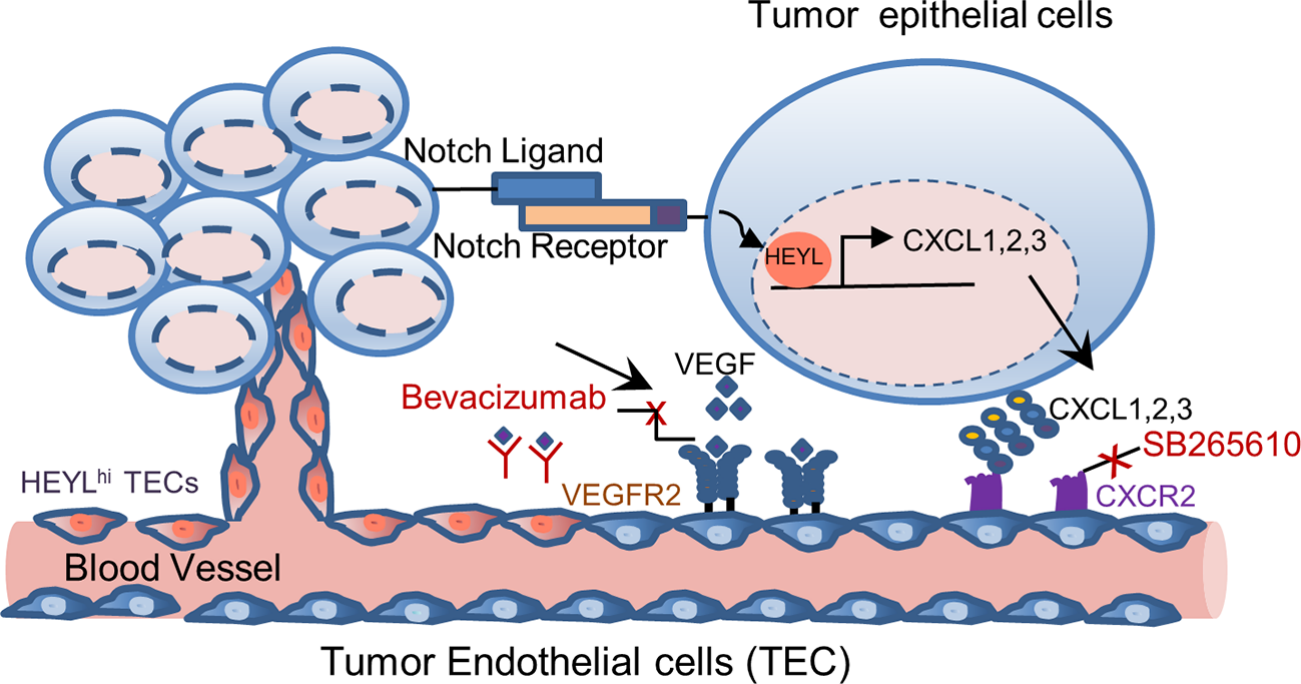
Model of differential functions of HEYL in tumor epithelial cells and endothelial cells. In carcinoma cells, HEYL is a known direct target of Notch. High HEYL transcriptionally upregulates expression of multiple angiogenic cytokines such as CXCL1, 2 and 3. Angiogenesis is enhanced by CXCL1, 2 and 3 binding to CXCR2, and VEGF binding to VEGFR2. Combined inhibition of these key molecules, CXCR2 and VEGF, achieves superior therapeutic benefits. In tumor endothelial cells, the expression of HEYL increases the endothelial cell invasion and thus promotes tumor angiogenesis.

In summary, we found that HEYL is expressed in both tumor epithelial and endothelial cells. HEYL exerts separable and potentially cooperating effects on angiogenesis. HEYL is a downstream target of Notch. Here, our data suggest a Notch-HEYL-angiogenic factor axis in breast cancer. HEYL may have angiogenic effects through overexpression in two different tumor cellular compartments. The complex and proposed functions of HEYL in tumor epithelial and endothelial cells are illustrated in **Figure 6**. Anti-HEYL targeted therapy may be a promising new component of treatment regimens aimed at targeting multiple angiogenic factors.

## Data Availability Statement

The datasets presented in this study can be found in online repositories. The names of the repository/repositories and accession number(s) can be found below: GSE161413, https://www.ncbi.nlm.nih.gov/geo/query/acc.cgi?acc=GSE161413.

## Ethics Statement

The animal study was reviewed and approved by Johns Hopkins Institutional Animal Care and Use Committee (JH-IACUC).

## Author Contributions

Conceptualization: LH, PK, SS. Project administration and methodology: SS, LH, PK, NN, AD, SC, WT, MG, LC, LR. Formal data analysis: LH, PK, SS, SC, LC. Writing—original draft: LH, Writing, review and editing. SS, LH, PK, LR, MG. All authors contributed to the article and approved the submitted version.

## Funding

This work was supported by the Department of Defense predoctoral fellowship W81XWH-04-1-0382 to LH, the DOD Center of Excellence Grant W81XWH-04-1-0595 and Susan G Komen Grant SAC110050 to SS.

## Conflict of Interest

The authors declare that the research was conducted in the absence of any commercial or financial relationships that could be construed as a potential conflict of interest.
