# Supplementary material for: HEYL Regulates Neoangiogenesis Through Overexpression in Both Breast Tumor Epithelium and Endothelium
Source: Front Oncol. 2021 Jan 15;10:581459. doi: 10.3389/fonc.2020.581459 (PMC7845423; doi:10.3389/fonc.2020.581459)
Supplement: Supplementary file 1 [file DataSheet_1.pdf]

Supplementary Materials

HEYL regulates neoangiogenesis through overexpression in both breast tumor epithelium and endothelium

Liangfeng Han[1], Preethi Korangath[1], Nguyen K Nguyen[1], Adam Diehl[1], Soonweng Cho[1], Wei Wen Teo[1], Leslie Cope[1], Manfred Gessler[2], Lewis Romer[3] and Saraswati Sukumar[1]

[1]Department of Oncology and [3]Departments of Anesthesiology and Critical Care Medicine, Cell Biology, Biomedical Engineering, and Pediatrics, Johns Hopkins University School of Medicine, [2]Developmental Biochemistry, Comprehensive Cancer Center Mainfraken and Theodor-Boveri-Institute/ Biocenter, University of Wurzburg, Wurzburg, Germany

saras@jhmi.edu

Supplementary Materials

Supp Table 1: pg 1-9

Supp Figure 1-2: pg 10

Supp Methods pg 11-13

References pg 13

Supplementary Table 1: Genes that showed over ≥2-fold expression changes at 6 and 24 hours after HEYL induction in HS578T-tet-off-HEYL inducible cells.

Probe set	Gene Symbol	6 Hour	24 Hour
209189_at	FOS	17.33961547	4.789914818
202768_at	FOS	17.26765178	3.160165247
202672_s_at	ATF3	12.13414036	8.357086103
1554980_a_at	ATF3	12.01695942	7.046954993
206115_at	EGR3	10.74058002	1.837825767
227404_s_at	EGR1	10.10501809	12.3976949
201694_s_at	EGR1	9.890202087	9.056791217
205249_at	EGR2	9.487315995	7.01770839
211506_s_at	IL8	7.983381752	1.014662547
1552721_a_at	FGF1	6.203145651	5.077083544
228442_at	NA	6.156028881	3.035638506
219270_at	CHAC1	5.848229794	6.272323633
211371_at	MAP2K5	5.771714747	7.658171666
210090_at	ARC	5.751746131	1.798756624
1555673_at	KRTAP2-1	5.567385567	4.101068952
238623_at	NA	5.544279543	2.220677667
223195_s_at	SESN2	5.318428433	6.611602545
206078_at	KALRN	5.091179741	9.113467018
209101_at	CTGF	5.010657754	2.438510188
201693_s_at	EGR1	5.007185835	8.562309587
216598_s_at	CCL2	4.900741328	1.263127262
223196_s_at	SESN2	4.89734557	7.695419637
209211_at	KLF5	4.779964819	3.375263185
202859_x_at	IL8	4.707626949	1.016070143
207536_s_at	TNFRSF9	4.688089135	4.688089135

Supp Table 1 (cont.)			
**Probe set**	**Gene Symbol**	**6 Hour**	**24 Hour**
210511_s_at	INHBA	4.525257851	1.53261996
222227_at	ZNF236	4.478451555	4.478451555
1560286_s_at	NA	4.429058338	3.258030252
220468_at	ARL14	4.263386944	3.358925972
1558404_at	LOC644242	4.210524619	2.352182501
204470_at	CXCL1	4.198866734	1.840375301
226991_at	NFATC2	4.193049902	2.267338826
210538_s_at	BIRC3	4.184339759	1.725482689
227140_at	NA	4.146804404	1.377450046
205599_at	TRAF1	4.036206535	3.358925972
242329_at	LOC401317	3.955883666	3.615010907
1555355_a_at	ETS1	3.877159268	2.128740365
207850_at	CXCL3	3.802636405	2.529759085
242625_at	RSAD2	3.797368484	2.124318373
204748_at	PTGS2	3.765913858	1.971098674
213418_at	HSPA6	3.660396673	1.798756624
1554997_a_at	PTGS2	3.61000291	2.039195366
209774_x_at	CXCL2	3.545526797	2.023706402
202887_s_at	DDIT4	3.501565319	3.1058755
205207_at	IL6	3.470154749	1.456999114
207526_s_at	IL1RL1	3.424753138	27.15225285
218182_s_at	CLDN1	3.379945538	2.572194967
220493_at	DMRT1	3.308093474	3.103723417
200800_s_at	HSPA1A	3.280691645	5.606109796
230372_at	NA	3.226567037	0.787853886
205681_at	BCL2A1	3.224331326	3.516158244

**Supp Table 1 (cont.)**			
**Probe set**	**Gene Symbol**	**6 Hour**	**24 Hour**
209212_s_at	KLF5	3.188769906	2.793357065
203889_at	SCG5	3.097276111	2.70007597
209305_s_at	GADD45B	3.095129987	4.996784503
222549_at	CLDN1	3.067365319	3.054634996
36711_at	MAFF	3.012580933	3.217633484
200796_s_at	MCL1	2.934266688	1.853176124
222771_s_at	MYEF2	2.930201749	4.900741328
205193_at	MAFF	2.922088757	3.151415544
206432_at	HAS2	2.907945035	0.660669203
243711_at	DDAH1	2.869899069	1.771535038
213797_at	RSAD2	2.850075228	2.289448321
200799_at	HSPA1A	2.846126922	3.755486989
205844_at	VNN1	2.816688454	1.587767862
236947_at	SEMA3C	2.772139771	1.059218335
205289_at	BMP2	2.762548896	0.947370071
207574_s_at	GADD45B	2.752991203	4.61394242
204472_at	GEM	2.747272467	2.661058082
220512_at	DLC1	2.73966596	1.309485423
214447_at	ETS1	2.681425183	1.559409685
205117_at	FGF1	2.674000991	2.928171392
201473_at	JUNB	2.64450921	1.727876375
237411_at	ADAMTS6	2.640845682	2.514026749
202149_at	NEDD9	2.622604028	3.673104649
209304_x_at	GADD45B	2.599078125	4.525257851
205659_at	HDAC9	2.588291309	2.080600533
204475_at	MMP1	2.582914701	5.087652027

Supp Table 1 (cont.)			
**Probe set**	**Gene Symbol**	**6 Hour**	**24 Hour**
202628_s_at	SERPINE1	2.579336501	0.889458994
207535_s_at	NFKB2	2.540301965	1.708819482
235745_at	ERN1	2.536782799	1.594384953
206157_at	PTX3	2.500124605	0.680185426
235737_at	TSLP	2.494931144	3.412904392
202393_s_at	KLF10	2.488023307	1.526259209
1554420_at	ATF3	2.488023307	3.206501318
230778_at	NA	2.4794154	1.714752073
205290_s_at	BMP2	2.475980582	0.893785162
202581_at	HSPA1B	2.463996147	2.954676127
201417_at	SOX4	2.443586203	1.4054187
204614_at	SERPINB2	2.435132037	1.834008086
202643_s_at	TNFAIP3	2.431758566	1.387992719
1560285_at	NA		1.851892045
234153_at	SYNJ2	2.418311352	0.84323111
205205_at	RELB	2.418311352	1.921189728
232676_x_at	MYEF2	2.404938498	4.38932775
207626_s_at	SLC7A2	2.348923942	0.589269704
1552972_at	hCG_2032978	2.347296357	1.778917987
229430_at	ADHFE1	2.344044567	1.950710923
215498_s_at	MAP2K3	2.337554497	1.273677475
231779_at	IRAK2	2.334316204	1.064370182
212614_at	ARID5B	2.313376368	3.333412829
221185_s_at	IQCG	2.30697121	2.234574276
201502_s_at	NFKBIA	2.300583787	1.139973273
1554960_at	C1orf110	2.281527432	0.458184322

Supp Table 1 (cont.)			
**Probe set**	**Gene Symbol**	**6 Hour**	**24 Hour**
228697_at	HINT3	2.278366754	2.358713185
230795_at	NA	2.261061134	3.986161051
203627_at	IGF1R	2.256364275	0.944092419
223394_at	SERTAD1	2.250116969	2.276788058
208394_x_at	ESM1	2.248557848	2.248557848
209239_at	NFKB1	2.243886961	1.094293701
225142_at	JHDM1D	2.234574276	3.657860358
208047_s_at	NAB1	2.226843236	0.942784536
202644_s_at	TNFAIP3	2.222217457	1.332374825
1556924_at	ALS2CR10	2.222217457	3.217633484
202880_s_at	PSCD1	2.20686748	1.264003098
201642_at	IFNGR2	2.188587403	0.930449658
224833_at	ETS1	2.182527754	1.336074078
235417_at	SPOCD1	2.177994031	1.247465572
236646_at	C12orf59	2.171963713	2.318191904
242907_at	GBP2	2.170458744	1.634670657
227458_at	NA	2.162949527	1.857033705
211139_s_at	NAB1	2.156960863	1.026689546
215499_at	MAP2K3	2.15248025	1.313121125
203665_at	HMOX1	2.148008943	2.121375483
226218_at	IL7R	2.146520573	3.750284386
204440_at	CD83	2.140577397	1.006257823
203927_at	NFKBIE	2.136130816	2.162949527
205807_s_at	TUFT1	2.133171562	3.417638964
227080_at	ZNF697	2.127265346	3.73471978
202269_x_at	GBP1	2.118436669	0.583579051

Supp Table 1 (cont.)			
**Probe set**	**Gene Symbol**	**6 Hour**	**24 Hour**
204994_at	MX2	2.118436669	1.708819482
219257_s_at	SPHK1	2.112571251	1.330529041
1557353_at	NA	2.095072254	1.178539408
210260_s_at	TNFAIP8	2.0907202	0.937571096
227755_at	NA	2.089271526	2.639015822
203153_at	IFIT1	2.086377187	2.295804828
202270_at	GBP1	2.084931522	0.557483109
203879_at	PIK3CD	2.083486858	1.572434584
214701_s_at	FN1	2.082043195	1.36983298
225803_at	FBXO32	2.070529848	2.318191904
203751_x_at	JUND	2.064797071	0.853817714
225516_at	SLC7A2	2.061936638	0.366275219
224219_s_at	TRPC4	2.054802879	2.536782799
240757_at	CLASP1	2.047693801	1.354724977
1556769_a_at	DLGAP1	2.046274939	2.791421528
214326_x_at	JUND	2.037782393	0.807201075
226533_at	HINT3	2.033549347	1.844206236
222802_at	EDN1	2.032140286	1.039579435
237732_at	NA	2.029325093	1.118061851
201615_x_at	CALD1	2.029325093	1.185914499
205239_at	AREG	2.02791896	4.263386944
225557_at	AXUD1	2.022304162	2.370185542
230499_at	BIRC3	2.019502595	0.927230546
1567224_at	HMGA2	2.018103268	0.576343173
242005_at	NA	2.008335086	1.910565873
210942_s_at	ST3GAL6	2.002774511	1.616641738

**Supp Table 1 (cont.)**			
**Probe set**	**Gene Symbol**	**6 Hour**	**24 Hour**
225842_at	PHLDA1	0.50243191	0.498615626
215058_at	MGC24039	0.499653546	0.340564509
241954_at	FDFT1	0.498270131	0.882702996
244503_at	BDNFOS	0.497579861	0.909408252
225239_at	NA	0.496890547	0.90312651
212240_s_at	PIK3R1	0.495858365	0.722966147
228523_at	NANOS1	0.492433221	0.545253866
239392_s_at	NA	0.492092011	0.410940094
239367_at	BDNF	0.490049708	0.880869374
228613_at	RAB11FIP3	0.487002134	0.738669032
224797_at	ARRDC3	0.484980955	1.208317843
227354_at	PAG1	0.484644908	0.644834125
203706_s_at	FZD7	0.484644908	0.788400174
218149_s_at	ZNF395	0.481964904	0.642157904
230192_at	TRIM13	0.475659138	0.78024548
222168_at	ALDH1A3	0.473685035	0.864537231
226034_at	NA	0.472701058	0.687770909
235926_at	ANAPC5	0.471065637	0.670821112
208937_s_at	ID1	0.466839972	1.511519928
224559_at	MALAT1	0.461691155	1.5888688
243438_at	PDE7B	0.461371246	0.461371246
235521_at	HOXA3	0.460732093	0.878430468
207069_s_at	SMAD6	0.45031299	0.835666959
230779_at	TNRC6B	0.446582926	0.77271055
230722_at	BNC2	0.431071773	0.423372656

Supp Table 1 (cont.)			
**Probe set**	**Gene Symbol**	**6 Hour**	**24 Hour**
227578_at	NA	0.430474594	0.737645729
212444_at	NA	0.421031477	0.496202187
229674_at	SERTAD4	0.408384496	0.581560021
208510_s_at	PPARG	0.400812665	0.796088099
236429_at	ZNF83	0.399425958	0.540362701
227062_at	TncRNA	0.396941965	0.645281245
235337_at	NA	0.390393246	0.473685035
227396_at	PTPRJ	0.387696603	0.523405141
202478_at	TRIB2	0.386087567	0.652929894
238478_at	BNC2	0.367037659	0.302918165
230494_at	SLC20A1	0.326691313	0.668500248
230109_at	PDE7B	0.287373712	0.408951029

Supp Table 1: Genes that showed over ≥2-fold expression changes at 6 and 24 hours after HEYL induction in HS578T-tet-off-HEYL inducible cells.

Supplementary Figures.

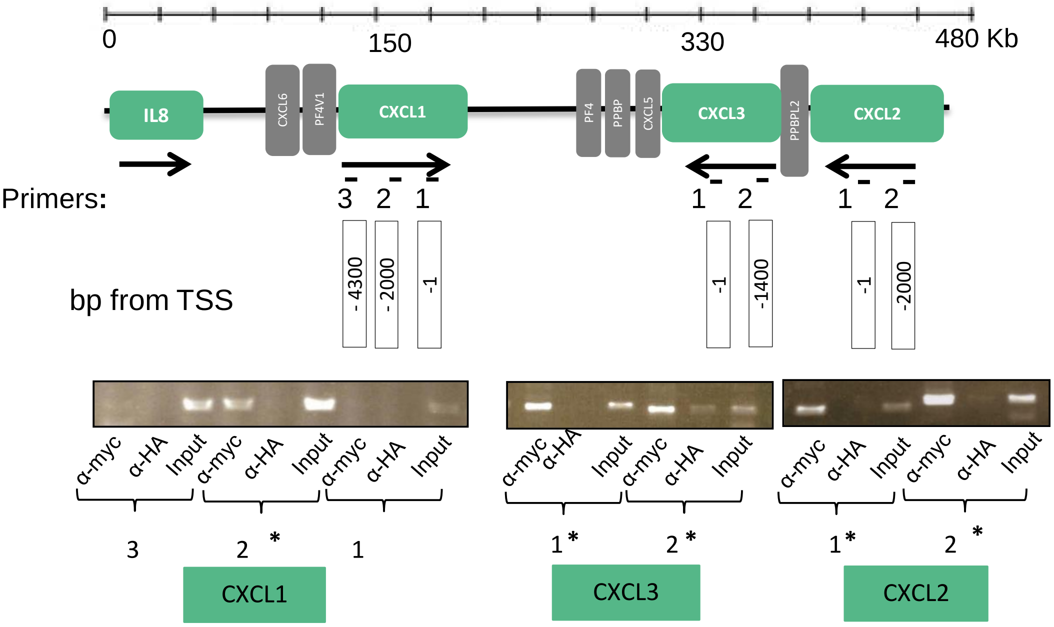

Supplementary Figure 1: ChIP analysis of putative HEYL-binding sites in CXCL1/2/3 promoter regions in HS578T-tet off-HEYL cells. Primers were designed to amplify seven predicted HEYL-binding sites (1) in CXCL1, 2 3 promoters (see Supp Methods for primer sequences). Among the seven predicted binding sites, five sites, denoted by asterisks were confirmed as HEYL-binding sites in breast cancer cells.

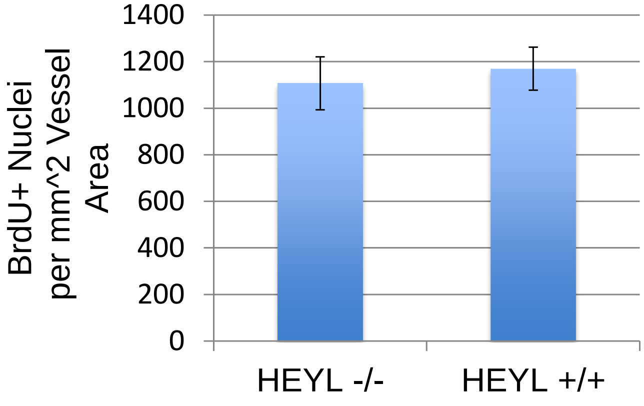

Supplementary Figure 2: Blood vessel endothelial cell proliferation in retina of HeyL+/+ or HeyL -/- mice as measured by in vivo BrdU incorporation assay.

Supplementary Methods.

Vascular characterization- quantitation of CD31-stained microvessels

Methods were followed as described (2) Formalin fixed paraffin-embedded sections of 2 mammary glands each from 5 mice were stained using the anti-CD31 antibody (Dianova). A 1:40 dilution of the antibody was used to stain the sections overnight. Diluted biotinylated anti-rat IgG (Vectastain kit) was added to the sections and incubated for 30 minutes. Vectastain ABC reagent (Vector) and 3, 30-diaminobenzamidine (DAB) was then used for color development. To capture differences between the vascularity of mammary glands taken from MMTV-HeyL transgenic mice compared to wild-type FVB/N mice, a single fourth mammary gland was taken from 13-week-old virgin HeyL transgenic mice (N=4) and virgin 13-week old wild-type mice (N=4). Sections of these mammary glands were prepared and scanned for the four most vascular fields within each mammary gland excluding the lymph node. Images of these four fields were captured with a Nikon Eclipse 50i camera with a PanFluor 20x objective using SPOT Advanced software. Within each field, the vessel number, cumulative circumferential vessel length and cumulative vessel area were quantified using MetaVue software by manually demarcating each vessel along its CD31 stained borders. These parameters were compared between virgin MMTV-HeyL transgenic mice and age-matched wild-type FVB/N mice using a Student's t test. The same protocol was also followed to study the MMTV-HeyL/Her2-neu (N=6) and Her2/neu (N=6) mammary tumor samples.

ChIP assay

HS578T-tet-off-HEYL cells on 150 mm tissue culture plates were uninduced or induced for 5 hours. Cells were fixed with 550 ul 37% formaldehyde for 6.5 min., lysed and the purified cell nuclei were sonicated for 6 min, 3 times (Jin, 2015). The sonicated DNA (500-1000 bp) was immunoprecipitated with 1.5 ul anti-HA or anti-Myc tagged HEYL antibody (Cell Signaling) using protein G magnetic ChIP assay bead kit (Millipore). The beads were extensively washed and DNA was eluted. The HEYL-binding sites at the different promoters were examined by PCR. The primer sequences used in CHIP assay are: CXCL1 transcription site 1, F: CGGGATCGATCTGGAACTC, R: GTGAGAGGAGCGGAAGAGC; CXCL1 transcription site 2, F: GTCTCCATTGGGTCAATGCT, R: GGTGTGCTAGAATGTTGTTTCTTT; CXCL1 transcription site 3, F: TCATTCTCTTGGCAGCTCCT, R: GCTGCCCAAATCTCTCATCT; CXCL2 transcription site 1, F: CTGGAGCTCCGGGAATTT, R: GAGGAGAGCTGGCAAGGAG; CXCL2 transcription site 2, F: GGATAGAAATGCACCCTCCTT, R: AAGCCTTATGAACACACACACG; CXCL3 transcription site 1, F: GGCTTTCCAGTCTCAACCAT, R: GGAAGCTGTGCGAGAAGC; CXCL3 transcription site 2, F: CTTGGGCTAGGCACAGAGAG, R: CCTGAAGCACAGGGCTCTAC.

HEYL adenovirus construction

HEYL-expressing adenovirus was constructed using published methods (He, 1998). HEYL cDNA was cloned into pAdtrack-CMV vector, cut with PmeI and introduced into electrocompetent E. coli BJ5183 cells with pAdEasy vector by electroporation. The adenoviral vectors were transfected into 293T cells and viral supernatant from the second round of infection was used for infection. In HUVEC cells, the adenovirus expressed HEYL and GFP from two different promoters. Adenovirus expressing GFP alone was used as control. Five ul of adenoviral supernatant was added into HUVEC cells cultured in a T75 flask (infection efficiency over 90% by GFP expression). The HUVEC cells were used for experiments one day later.

Retinal vessel staining

The techniques were essentially as described in (3). Six-day old Heyl +/+ and HeyL -/- mouse pups (littermates from the same cage) were used for comparison. A freshly prepared stock (3 mg/ml) of 5′-bromo-2′ deoxyuridine (BrdU, Invitrogen, cat. no. B23151) was used each time; 300 µg of BrdU was injected intraperitoneally (i. p.) into the 6-day-old pups. 2.5 h later the mice were euthanized and the eyes fixed in 4% paraformaldehyde (PFA) at 4oC overnight, washed in PBS and retina was dissected out. After blocking/permeabilization in 1% After blocking/permeabilization in 1% BSA with 0.3% triton o. n at 4oC, the retina was washed two times in Pblec buffer (1% Triton X100, 1mM CaCl2, 1mM MgCl2 and 1mM MnCl2 in PBS, pH 6.8) for 20 min and incubated for 2 h in PBS containing biotinylated isolectin B4 (Vector Labs 1:50) (4, 5). Following five washes (each 20 min) in blocking solution, the retina was incubated with Alexa-Fluor-streptavidin-conjugated antibodies (Molecular Probes, 1:100) for 2 h. After washing three times in PBS, the retina was re-fixed in 4% PFA-PBS for 30 min at RT. The retinas were washed for 5 min in 2 ml of PBS at RT and incubated in 2 ml of formamide-SSC solution for 1 h at 65oC. The formamide-SSC solution was removed completely and retina incubated in 2ml of 2N HCl solution for 30 min at 37□C. The 2N HCl solution was removed completely and retina neutralized by washing twice (10min/ wash) with 2 ml of 0.1M Tris HCl (pH 8.0) at RT. The solution was removed and retina washed twice (10 min/wash) with 2 ml of PBS at RT. The retina was incubated with 2 ml of retina-blocking buffer for 2 h at RT. While the retina was being treated, monoclonal mouse anti-BrdU antibody (BD Bioscience, cat. no. 347580) at a dilution 1:50 was diluted in retina-blocking buffer and 2% (V/V) goat serum. The blocking solution was removed completely and the retina incubated overnight at 40 □C with 100 ul of diluted mouse anti-BrdU antibody. Retinas were washed five times (20 min per wash) using 2 ml of wash buffer at RT. While the retinas were being washed, the secondary goat anti-mouse antibody (Alexa Fluor 546, dilution 1:500) was diluted in retina blocking buffer and 2% (V/V) goat serum. The washing buffer was removed and 100 ul of secondary antibody solution was added to the retinas and incubated for 2 h at RT. The retinas were washed four times (20 min per wash) with 2 ml of wash buffer at RT, and flat-mounted on microscope glass slides with Fluoromount-G (SouthernBiotech, 0100-01).

After staining and flat mounting of retinas, images were taken using a Hamamatsu C10600 camera attached to a Nikon Eclipse TE200 microscope. For each magnification level, the gain and exposure time was held constant across all images taken. Volocity Software was used for image capture and contrast enhancement, which was done uniformly across all images. Image J was used for analysis.

The retina was flattened with either four or five incisions radially around the optic nerve. Therefore, each retina had four or five wedges extending from the optic nerve along which measurements of retinal vessel migration distance could be made. Eleven retinas from 7 HeyL- /- mice and 8 retinas from 5 HeyL+/+ mice were examined. 4x images (2738 um x 2086 um) were taken of each of the 4 or 5 retinal wedges and Image J software was used to quantify the length of a line segment bisecting the wedge and extending from the outer margin of the optic nerve to the border of the vascular network. The average length from the optic nerve to the vascular front was calculated for the HeyL-/- mice and the HeyL+/+ mice and the two were compared using 2-tailed Student's T test.

For quantification of BrdU positive endothelial cells, 2 retina samples were taken from each of three HeyL-/- mice (n=6) and three HeyL+/+ mice (n=6). These retinas were physically cut and flattened into four quadrants, and 10x images (1095 um x 835 um) were taken of each of the four quadrants. For quantification of the number of BrdU positive cells per square millimeter of endothelial cell area coverage, we focused on the vascular front, an area defined by the width of

the quadrant and a height equal to the most distal 250 um of retinal vessel expansion away from the optic nerve. Using Image J, this area was manually traced, and the number of BrdU positive cells lying in this region was manually counted. The vessel area was calculated using Image J to select the strongly fluorescent region within the defined area. The BrdU positive cell count was then normalized to the vessel area and these values were averaged for the two groups and compared using a 2-tailed Student's T-test.

References.

1. Heisig, J, Weber, D, Englberger, E, Winkler, A, Kneitz, S, Sung, WK, et al. Target gene analysis by microarrays and chromatin immunoprecipitation identifies HEY proteins as highly redundant bHLH repressors. PLoS genetics. 2012;8(5):e1002728. doi: 10.1371/journal.pgen.1002728. PubMed PMID: 22615585; PMCID: PMC3355086.
2. Wu, FT, Paez-Ribes, M, Xu, P, Man, S, Bogdanovic, E, Thurston, G, et al. Aflibercept and Ang1 supplementation improve neoadjuvant or adjuvant chemotherapy in a preclinical model of resectable breast cancer. Sci Rep. 2016;6:36694. Epub 2016/11/15. doi: 10.1038/srep36694. PubMed PMID: 27841282; PMCID: PMC5107907 from Regeneron. R.S.K. is a member of Scientific Advisory Board of Angiocrine Bioscience Inc. and MolMed Inc., a consultant to Triphase Accelerator LLC, and has received honoraria recently from Boehringer-Ingelheim, Eli Lilly and Neovacs Pharma.
3. Pitulescu, ME, Schmidt, I, Benedito, R, Adams, RH. Inducible gene targeting in the neonatal vasculature and analysis of retinal angiogenesis in mice. Nat Protoc. 2010;5(9):1518-34. Epub 2010/08/21. doi: 10.1038/nprot.2010.113. PubMed PMID: 20725067.
4. Higuchi, M, Kato, T, Yoshida, S, Ueharu, H, Nishimura, N, Kato, Y. PRRX1- and PRRX2-positive mesenchymal stem/progenitor cells are involved in vasculogenesis during rat embryonic pituitary development. Cell Tissue Res. 2015;361(2):557-65. Epub 2015/03/22. doi: 10.1007/s00441-015-2128-5. PubMed PMID: 25795141.
5. Benton, RL, Maddie, MA, Minnillo, DR, Hagg, T, Whittemore, SR. Griffonia simplicifolia isolectin B4 identifies a specific subpopulation of angiogenic blood vessels following contusive spinal cord injury in the adult mouse. J Comp Neurol. 2008;507(1):1031-52. Epub 2007/12/20. doi: 10.1002/cne.21570. PubMed PMID: 18092342; PMCID: PMC2735010.
